# Supplementary material for: Properties of Liquid Crystalline Elastomer Foams
Source: Macromol Rapid Commun. 2026 Aug 6;47(18):e70395. doi: 10.1002/marc.70395 (PMC13592172; doi:10.1002/marc.70395)
Supplement: Supplementary file 1 [file MARC-47-e70395-s001.docx]

Supporting Information

Properties of Liquid Crystalline Elastomer Foams

Oliver Dai, Andrew Terentjev, and Eugene M. Terentjev*

**S1: Chemicals used for LCE synthesis**

*Figure S1: Chemical structure of (a) 1,4-bis-[4-(3-acryloyloxypropypropyloxy) benzoyloxy]-2 methylbenzene (RM257); (b) 2,2’-(ethylenedioxy) diethanethiol (EDDT); (c) pentaerythritol tetrakis (3-mercaptopropionate) (PETMP); (d) Triphenylphosphine (TPP); (e) butylated hydroxytoluene (BHT, from Sigma Aldrich). The RM257:EDDT:PETMP:TPP:BHT ratio is 20:14:2:2.5:1.*

**S2: Nouryon 920DU40 physical structure**

*Figure S2: As received Nouryon 920DU40 Expancel Microspheres powder has an average diameter of 12 µm and a shell thickness of 2 µm. On heating at T = 160°C, the liquid blowing agent expands the ECM sphere, with sphere diameter growing to 40 µm and shell thickness thinning to 0.1 µm. Previous tests by Nouryon have shown that the microsphere surfaces* $\gamma\sim50 \text{mN/m}$.

**S3: Deformation of ECM under extensive strain**

*Figure S3: ECM-loaded LCE foam, (a) post-cured, before stretching; (b) after manual stretching by hand. The elasticity of Expancel microspheres allows the Eshelby theory for simple elastic inclusions to be applicable.*

**S4: Relating desired foaming volume fraction to ECM weight fraction in curing mixture**

**Densities of ECM before and after expansion**

The unexpected ECM density is given by the formula below:

$$\rho_{i}=\frac{m_{T}}{V_{T}}=\frac{\rho_{acrylate}\left( V_{T}-V_{blow} \right)+\rho_{blow}V_{blow}}{V_{T}}$$

where $V_{T}$ and $m_{T}$ are the overall ECM volume and mass, respectively, $\rho_{acrylate}$ is the acrylate copolymer density, $\rho_{blow}$ is the blowing agent density, and $V_{blow}$ the blowing agent volume. Assuming that the blowing agent density is low, we can write:

$$\rho_{i}=\rho_{acrylate}(1-{V_{blow}}/{V_{T}})$$

Using the proportionality relationship $V\propto r^{3}$, we obtain

$$\rho_{i}=\rho_{acrylate}\left( 1-\left( {r_{blow}}/{r_{T}} \right)^{3} \right)$$

Substituting the values given above in section S2 ($\rho_{acrylate}=1.1 {g cm}^{-3}, r_{blow}={4 \mu m , r}_{T}=6 \mu m$) gives:

$$\rho_{i}=0.774 {g cm}^{-3}$$

By conservation of mass,

$$\rho_{f}=\rho_{i}\frac{V_{i}}{V_{f}}=\rho_{i}{(\frac{r_{i}}{r_{f}})}^{3}$$

where $\rho_{f}$, $r_{f}$, and $V_{f}$ are the expanded ECM density, outer radius, and volumes, respectively, and $r_{i}$ the unexpanded ECM outer radius. Evaluation gives

$\rho_{f}=0.021 {g cm}^{-3}$.

**Relating mixture weight fraction of ECM to observed volume fraction**

The weight fraction of ECM, $M$, can be related to the observed volume fraction of ECM, $R_{V}$, via:

$$R_{V}=\frac{V_{ECM}}{V_{LCE}+V_{ECM}}=\frac{{m_{ECM}}/{\rho_{ECM}}}{{m_{ECM}}/{\rho_{ECM}}+{m_{LCE}}/{\rho_{LCE}}}$$

where $V_{ECM}$, $m_{ECM}$ and $\rho_{ECM}$ are the ECM volume, mass, and densities, respectively, and $V_{LCE}$, $m_{LCE}$ and $\rho_{LCE}$ the LCE volume, mass, and densities, respectively. This rearranges into:

$$R_{V}=\frac{1}{1+{\rho_{ECM}}/{(\rho_{LCE}\times M)}}$$

where $\rho_{ECM}=\rho_{f}=0.021 {g cm}^{-3}$ and $\rho_{LCE}=1.2 {g cm}^{-3}$.

**S5: ECM weight fractions added to achieve the desired volume fractions**

| **Sample label** | ECM mass added [mg] | ECM  weight fraction (M) [%] | Expanded ECM  volume fraction $R_{V}$ [%] |
| --- | --- | --- | --- |
| LCE 0 | 0 | 0 | 0 |
| LCE 0.25 | 0.9 | 0.004 | 0.25 |
| LCE 0.6 | 2.3 | 0.01 | 0.62 |
| LCE 1.2 | 4.6 | 0.02 | 1.2 |
| LCE 1.8 | 7.0 | 0.03 | 1.8 |
| LCE 2.5 | 9.3 | 0.04 | 2.5 |
| LCE 3.6 | 16 | 0.07 | 3.6 |
| LCE 5 | 21 | 0.09 | 4.7 |
| LCE 9 | 40 | 0.17 | 9.0 |
| LCE 13 | 60 | 0.26 | 13 |

**Supporting Information**

Supporting Information is available from the Wiley Online Library or from the author.
